# Long-standing, multifocal Tuberculosis verrucosa cutis: Undiagnosed for 40 years

**DOI:** 10.1016/j.idcr.2026.e02568

**Published:** 2026-04-06

**Authors:** Seblewengel Maru Wubalem, Helina Melis Kefetew, Saada Yasin Abduselam, Shemsu Abraham Hussien

**Affiliations:** a Department of Pathology, Wachemo University, Hossana, Ethiopia; b Department of Dermatology, Wachemo University, Hossana, Ethiopia

**Keywords:** Cutaneous tuberculosis, Tuberculosis verrucosa cutis, Long standing CTB, FNAC

## Abstract

**Background:**

Tuberculosis (TB) is a chronic infectious disease primarily affecting the lungs, with extrapulmonary TB comprising 20% of cases. Among these, cutaneous TB (CTB) accounts for 1–2%, often overlooked due to its rarity and varied presentations. This report highlights an unusual case of Tuberculosis verrucosa cutis (TVC)

**Case presentation:**

A 60-year-old male, presented with non-healing skin lesion that remained undiagnosed for 40 years. The lesion, which involved multiple areas of the body, exhibited large annular plaques with indurated edges and atrophic scars. Diagnosis was made through a detailed clinical history and examination, confirmed by fine-needle aspiration cytology (FNAC), which revealed necrotizing granulomatous inflammation. The patient responded well to anti-TB treatment, showing significant improvement within two months.

**Conclusion:**

This case underscores the importance of clinical suspicion in diagnosing CTB, particularly in endemic regions, and suggests that FNAC is a valuable diagnostic tool in resource-limited settings.

## Highlights

• This report highlights Tuberculosis verrucosa cutis (TVC) that remained undiagnosed for 40 years.
• TVC is exogenous form of cutaneous tuberculosis (CTB).
• CTB may go undiagnosed for several years due to its rarity and diverse presentation.
• FNAC might be helpful in diagnosing CTB, especially in resource limited setups.

## Introduction

Tuberculosis (TB) is a chronic infectious disease caused by *Mycobacterium tuberculosis (Mtb)* that primarily affects the lungs. It is a significant public health problem, with a higher incidence in developing countries. Extrapulmonary TB accounts for 20% of cases, among which cutaneous TB (CTB) represents 1–2% [1], [2]. CTB is frequently overlooked due to its rarity and diverse presentations. Diagnosing CTB necessitates a high level of clinical suspicion. In this report, we present an unusual form of CTB, Tuberculosis verrucosa cutis (TVC), which remained undiagnosed for 40 years.

## Case presentation

A 60-year-old male patient presented to our dermatology outpatient clinic with a complaint of a non-healing, slowly progressive skin lesion of over forty years' duration. The lesion initially appeared in the right axilla and gradually extended to other areas, including both sides of the neck, the right arm, and the forearm. He reported mild, occasional itching and some restriction in extending his right forearm. The patient mentioned traveling to a remote town a few months before the onset of the lesion but denied any history of TB diagnosis or treatment, and he did not recall contact with anyone who had been treated for TB. Otherwise, he has no known chronic medical illnesses such as hypertension, diabetes mellitus, or HIV. He had tried various unspecified herbal, over-the-counter, and prescribed topical medications without improvement. He got the prescribed medications from private clinics for the diagnoses that he could not specify.

**Fig. 1:**
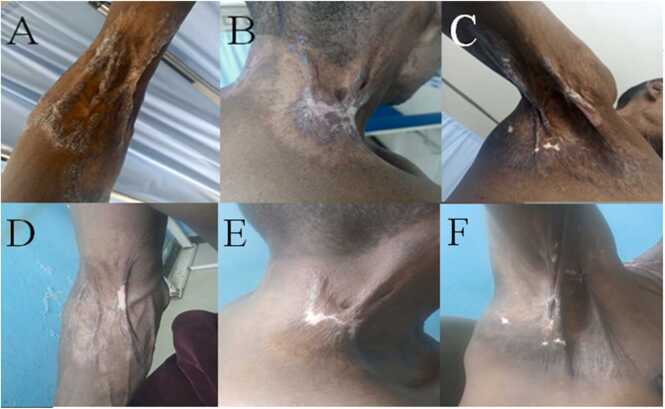
Tuberculosis verrucosa cutis. Prior to treatment, annular plaques are visible on the elbow, neck, and axilla (A, B, and C); following anti-tuberculosis therapy, significant clinical improvement is noted, with only scarring and hypopigmentation remaining (D, E, and F).

On skin examination; multiple large annular plaques were observed, each measuring approximately 15–20 cm in diameter. These plaques were located on both sides of the neck, in the right axilla, and around the right elbow, extending to the arm and forearm. The edges of the lesions were indurated, with overlying scales and adherent brownish crusts. The centers displayed atrophic scars with linear ridges of hypertrophic scarring, which caused some limitation in the range of motion (Fig. 1).

Baseline investigations—including complete blood count, erythrocyte sedimentation rate, and organ function tests—were within normal limits, and chest X-ray findings were unremarkable. Fine-needle aspiration cytology (FNAC) from the neck, elbow, and axillary lesions revealed numerous lymphocytes, epithelioid granulomas, multinucleated giant cells, and necrosis. This led to a final impression of necrotizing granulomatous inflammation consistent with TB. Acid-fast bacilli (AFB) stain and GeneXpert testing were negative. A skin biopsy was deferred as the clinical and cytological findings were diagnostic of TVC.

The patient was started on anti-TB therapy. After two months, significant improvement was noted with near-total clearance of the lesions. Upon completion of the full anti-TB regimen, the lesions had cleared, leaving only residual hypopigmentation and scarring (Fig. 1). While the patient reported that all other symptoms had subsided, he experienced limited range of motion at the scar sites. He has since been referred to the surgical department for plastic surgery intervention to manage the linear hypertrophic scars.

## Discussion

CTB presents with a variety of clinical forms, including inflammatory papules, verrucous plaques, suppurative nodules, chronic ulcers, and other atypical lesions [2]. It is categorized according to the route of infection—exogenous, endogenous, and hematogenous—and the pathogen load present in the skin [2], [3]. Exogenous CTB occurs through the introduction of the pathogen into the skin via traumatic injuries, contaminated surgical procedures, or body piercings. Tuberculous chancre and TVC are examples of exogenous CTB [2], [4], [5].

Endogenous CTB may arise from the contiguous infection of skin overlying a subcutaneous focus, such as TB lymphadenitis, osteomyelitis, or epididymitis, commonly known as scrofuloderma. Another type of endogenous infection is TB cutis orificialis, which results from autoinoculation of the mucous membranes surrounding body orifices [2], [4].

Forms of CTB such as lupus vulgaris, metastatic tuberculous abscess, and acute miliary TB can spread through the bloodstream. These lesions develop when the mycobacterium disseminates from the primary site of infection to the skin via hematogenous routes. CTB is further classified based on pathogen load as either multibacillary or paucibacillary [3], [4].

TVC is a rare form of paucibacillary cutaneous TB (CTB), accounting for 6% of all CTB cases. It is caused by exogenous reinfection in an immune-sensitized host. Inoculation occurs through wounds and occasionally from the patient's own sputum [6], [7]. This form of TB is associated with occupational exposure in adults, such as pathologists, laboratory personnel, and farmers [6], [8]. It can affect the fingers, hands, ankles, buttocks, and lower extremities in children. Children acquire the infection by walking barefoot on ground contaminated with TB sputum [6], [9].

TVC presents as a solitary, slowly enlarging red-brown papule. It may develop into a verrucous plaque that resembles a typical wart, characterized by clefts and fissures on its surface. As it progresses, it can grow larger through peripheral expansion, which may be accompanied by central atrophy or ulceration [4], [8]. There are just over a dozen reported cases of multifocal TVC [10]. Our case exhibits the typical appearance, with multifocal lesions. This condition can remain undiagnosed for extended periods, sometimes up to 60 years, with an average duration of 10 years, as patients typically do not show systemic symptoms and the lesion can mimic various verrucous skin conditions [8]. In this case, the lesion went undiagnosed for 40 years. The patient did not exhibit any systemic symptoms and sought medical attention multiple times for the skin lesion, receiving various topical treatments without improvement.

CTB is a difficult form of extrapulmonary TB to diagnose, as it shows low sensitivity and specificity for many diagnostic methods [11]. A detailed clinical history and physical examinations, along with laboratory workups, are crucial for reaching the diagnosis. These workups include the demonstration of bacilli by AFB stain, tuberculin skin test, culture, molecular studies, and histology. Culture is considered the gold standard for diagnosis. Molecular diagnostic methods, such as polymerase chain reaction (PCR), are useful for detecting the infection and assessing drug sensitivity [12].

The histological features of TVC consist of pseudoepitheliomatous hyperplasia of the epidermis with hyperkeratosis and a dense inflammatory cell infiltrate, including neutrophils, lymphocytes, and giant cells [13]. AFB stains, as well as culture and PCR, are often negative in paucibacillary CTB like TVC. In such cases, a treatment trial is used as an alternative method to confirm the diagnosis [7], [12].

In our patient, there was a strong clinical suspicion of CTB, supported by FNAC findings that demonstrated a necrotic background and granulomas. Therefore, the patient was initiated on anti-TB treatment and showed significant improvement within two months, which supports the diagnosis of TVC. Although FNAC is not commonly mentioned as a diagnostic tool for CTB in many literatures, we found it helpful and suggest its utilization, especially in resource-limited settings like ours.

CTB is treated with rifampicin, isoniazid, ethambutol, and pyrazinamide. The intensive phase lasts two months, followed by a maintenance phase of four months [1], [12], [14]. CTB patients typically respond within 4–6 weeks of treatment, unless there is drug resistance or a misdiagnosis [12]. Scar formation with wound contracture is one of the complications of long-standing CTB. In such cases, surgical management plays a role [14]. In this case, the patient responded to anti-TB treatment with significant improvement in two months. However, he developed a wound contracture with a limitation of range of motion; therefore, it is planned to refer him to the surgical department.

## Conclusion

CTB can remain undiagnosed for years. A strong clinical suspicion, especially in areas where TB is prevalent, is a key. Many diagnostic workups have low sensitivity and specificity for CTB. FNAC is a helpful diagnostic modality in resource-limited settings. CTB responds well to the standard anti-TB regimen.

## CRediT authorship contribution statement

**Saada Yasin Abduselam:** Writing – review & editing, Data curation, Conceptualization. **Helina Melis Kefetew:** Writing – review & editing, Data curation, Conceptualization. **Seblewengel Maru Wubalem:** Writing – review & editing, Writing – original draft, Supervision, Data curation, Conceptualization. **Shemsu Abraham Hussien:** Writing – review & editing, Data curation, Conceptualization.

## Patient consent statement

Written informed consent was obtained from the patient for publication of this case report and accompanying images.

## Ethics approval

The study was notified to the university ethics committee; but this is a case report and it does not need a specific ethical approval.

## Funding

This work did not receive any specific grant from funding agencies in the public, commercial, or not-for-profit sectors*.*

## Declaration of Competing Interest

The authors declare that they have no known competing financial interests or personal relationships that could have appeared to influence the work reported in this paper.

## References

[1] Brito A.C., Oliveira C.M.M., Unger D.A., Bittencourt M.J.S. (2022). Cutaneous tuberculosis: epidemiological, clinical, diagnostic and therapeutic update. Bras De Dermatol.

[2] Khadka P., Koirala S., Thapaliya J. (2018). Cutaneous tuberculosis: clinicopathologic arrays and diagnostic challenges. Dermatol Res Pract.

[3] Hill, M.K., & Sanders, C.V. (2017). Cutaneous Tuberculosis. Microbiol Spectr, 5(1), 10.1128/microbiolspec.tnmi7-0010-2016. 10.1128/microbiolspec.TNMI7-0010-2016. PMC11687432 28233513

[4] Nguyen K.H., Alcantara C.A., Glassman I., May N., Mundra A., Mukundan A., Urness B., Yoon S., Sakaki R., Dayal S., Chowdhury T., Harshavardhan S., Ramanathan V., Venketaraman V. (2023). Cutaneous manifestations of *Mycobacterium tuberculosis*: a literature review. Pathog (Basel Switz).

[5] Semaan R., Traboulsi R., Kanj S. (2008). Primary mycobacterium tuberculosis complex cutaneous infection: report of two cases and literature review. Int J Infect Dis IJID Publ Int Soc Infect Dis.

[6] Aliagaoglu C., Atasoy M., Gulec A.I., Ozdemir S., Erdem T., Karakuzu A., Aktas A., Timur H., Engin R.I. (2007). Tuberculosis verrucosa cutis. Experience from eastern Turkey. Saudi Med J.

[7] Pebriany D., Anwar A.I., Djamaludin W., Adriani A., Amin S. (2020). Successful diagnosis and management of tuberculosis verrucosa cutis using antituberculosis therapy trial approach. Pan Afr Med J.

[8] Santos J.B., Figueiredo A.R., Ferraz C.E., Oliveira M.H., Silva P.G., Medeiros V.L. (2014). Cutaneous tuberculosis: epidemiologic, etiopathogenic and clinical aspects - part I. Bras De Dermatol.

[9] Wedy G.F., Passero L.F.D., Criado P.R., Belda W. (2021). A case of tuberculosis verrucosa cutis in Brazil undiagnosed for 15 years. Braz J Infect Dis Publ Braz Soc Infect Dis.

[10] Ntavari N., Syrmou V., Tourlakopoulos K., Malli F., Gerogianni I., Roussaki A.V., Zafiriou E., Ioannou M., Tziastoudi E., Gourgoulianis K.I., Pantazopoulos I. (2023). Multifocal tuberculosis verrucosa cutis: case report and review of the literature. Med (Kaunas Lith).

[11] Costa L.L., Veasey J.V. (2021). Diagnosis of cutaneous tuberculosis (lymph node scrofuloderma) using the Xpert MTB/RIF® method. Bras De Dermatol.

[12] Panchatsharam A., Sundaramurthy R. (2023). Cutaneous tuberculosismanagement. Int J Res Dermatol.

[13] Xiao Yu, Peng Shan-Shan, Zhang Lyu-Ya, Lin Jie, Hu You-Hong, Fang Mu-Ping (2023). Tuberculosis Verrucosa Cutis on the Buttocks: A Case Report. Int J Dermatol Venereol.

[14] Soedjana H., Riestiano B.E., Hasibuan L.Y., Harianti S. (2024). Management of cutaneous tuberculosis in hand - rare and disabling: a case report. Int J Surg case Rep.

